# Markers of Disease Severity in Patients with Spanish Influenza in the Japanese Armed Forces, 1919–1920

**DOI:** 10.3201/eid2304.152097

**Published:** 2017-04

**Authors:** Koichiro Kudo, Toshie Manabe, Shinyu Izumi, Jin Takasaki, Yuji Fujikura, Akihiko Kawana, Kenji Yamamoto

**Affiliations:** Koto Hospital, Tokyo, Japan (K. Kudo);; Waseda University Organization for Regional and Inter-regional Studies, Tokyo (K. Kudo, T. Manabe);; Teikyo University School of Medicine, Tokyo (T. Manabe);; Tsukuba University Graduate School of Comprehensive Human Sciences, Ibaraki, Japan (T. Manabe);; National Center for Global Health and Medicine, Tokyo (S. Izumi, J. Takasaki, K. Yamamoto);; National Defence Medical College, Saitama, Japan (Y. Fujikura, A. Kawana);; So-Yu Medical Foundation Institute of Medicine, Tokyo (K. Yamamoto)

**Keywords:** Spanish influenza, respiratory illnesses, pneumonia, viral pneumonia, bacterial pneumonia, adventitious lung sounds, time course of fever, respiration rate, length of hospitalization, disease severity, second wave, influenza virus, influenza pandemic, viruses, respiratory infections, zoonoses, Japan

## Abstract

We examined preserved medical charts of 470 Spanish influenza patients (8 with fatal cases) hospitalized at former army hospitals in Japan during 1919–1920. The following factors were associated with longer periods of hospitalization: adventitious discontinuous lung sounds, maximum respiration rate, continuation of high fever after hospital admission, and diphasic fever.

The first and second waves of the Spanish influenza pandemic in Japan affected ≈21 million persons (257,000 deaths) and 2 million persons (127,000 deaths), respectively (*1*). Although available clinical techniques and treatment options for Spanish influenza patients were limited in this era, charts showing detailed records of lung sounds and fever exist and can be used to infer disease severity in affected persons. We aimed to identify physical features, including respiratory sounds, that might be associated with disease severity among patients in Japan who were affected by Spanish influenza during 1919 and 1920.

## The Study

We analyzed medical charts preserved at the former First Army Hospital in Tokyo, Japan, and other affiliated hospitals. We previously described the clinical features of Spanish influenza among patients who were hospitalized at several study sites (*2*). Recently, additional records of patients affected by the second wave of disease during 1919–1920 were discovered, and these patients were the subjects of this study. 

A total of 470 patients hospitalized during January 1919–January 1920 and diagnosed with Spanish influenza (as “epidemic cold” or “pneumonia due to epidemic cold”) fit the criteria for inclusion in the study. All patients were male soldiers or officers in the military of Japan. We collected data concerning patients’ general background and physical assessments, including lung sounds and fever charts. Among all patients, 8 (2%) died. We divided the patients who survived (n = 462, 98%) into 3 groups on the basis of hospitalization length: <10 days (28%), 11–20 days (34%), and >21 days (36%); we compared variables among the 3 groups. High fever was defined as a body temperature >38°C, and diphasic fever was defined as a body temperature >38°C after the initial fever had decreased to <37.5°C. Data on adventitious lung sounds collected during the hospitalization period were classified (on the basis of international classifications) as continuous, discontinuous, bronchial on the chest wall, and friction rub sounds (*3*). The study was approved by the Institutional Review Board of the National Center for Global Health and Medicine, Tokyo, Japan.

Of the 8 patients who died, 6 died within 10 days of hospital admission. Median length of hospitalization was 7 days for nonsurvivors and 16 days for survivors. The proportion of patients with audible adventitious lung sounds was significantly higher among those hospitalized for >21 days and among those who did not survive (Table 1). Factors associated with the length of hospitalization in survivors (identified by using a Cox hazard proportional model) included diphasic fever, >6 days of continuing high fever from admission, a maximum respiration rate >26 breaths/min, and adventitious discontinuous lung sounds (Table 2).

**Table 1:** General characteristics and clinical findings of patients with Spanish influenza during hospitalization, Japan, 1919–1920*

Characteristic/clinical feature	Nonsurvivors, n = 8	Survivors, by hospitalization length, n = 462			
<10 d, n = 131	11–20 d, n = 161	>21 d, n = 170	p value†
Median age, y (IQR)	22 (21–23)	22 (21–28)	21 (20– 27)	21 (20–22)	<0.001‡
Time from onset to first visit, median d (IQR)	2 (1 – 3)	1 (1–1)	1 (0 – 1)	1 (1–2)	0.081‡
Hospitalization, median d (IQR)	7 (6 −14)	8 (6–9)	15 (13 – 17)	32 (25–40)	<0.001‡
Duration of high fever from admission, median d (IQR)	7 (5–14)	3 (2–4)	7 (5 – 8)	10 (7–18)	<0.001‡
>6 d, no. (%)	6 (75.0)	12 (9.2)	91 (56.5)	123 (72.8)	<0.001§
Maximum respiration rate during hospitalization, median (IQR)	53 (45–60)	24 (21–26)	25 (24–30)	29 (25–33)	<0.001‡
≥26 breaths/min., no. (%)	8 (100.0)	10 (16.4)	34 (39.5)	74 (65.5)	<0.001§
Diphasic fever, no. (%)	0	12 (9.2)	51 (31.7)	111 (65.7)	<0.001§
Adventitious lung sounds, no. (%)					
Discontinuous	8 (100.0)	45 (34.4)	96 (59.6)	141 (82.9)	<0.001§
Continuous	8 (100.0)	75 (57.3)	121 (75.2)	144 (84.7)	<0.001§
Bronchial sounds on chest wall	5 (62.5)	7 (5.3)	13 (8.1)	27 (15.9)	0.002¶
Friction rub	2 (25.0)	2 (1.5)	6 (3.7)	21 (12.4)	<0.001¶
Clinical symptoms, no. (%)					
Upper respiratory tract#	7 (87.5)	126 (96.2)	155 (96.3)	157 (92.4)	0.071§
Dyspnea/tachypnea	8 (100.0)	108 (82.4)	144 (89.4)	159 (93.5)	0.003§
Gastric intestinal	5 (62.5)	26 (19.8)	41 (25.5)	61 (35.9)	0.001§
Psycho/mental**	7 (87.5)	121 (92.4)	150 (93.2)	153 (90.0)	0.273§
*High fever, body temperature of >38°C; diphasic fever, body temperature >38°C at a time after the initial fever had decreased to <37.5°C. IQR, interquartile range; †p values were calculated for the 3 groups on days of hospitalization of survivors (<10 d, vs 11 – 20, vs >21 d). ‡By Kruskal-Wallis test. §By χ^2^ test. ¶By Fisher exact test. #Upper respiratory tract symptoms were cough, sputum, and wheezing. **Psycho/mental denotes psychological and mental disturbances, including anxiety, irritation, delirium, and confusion.					

**Table 2:** Risk factors for length of hospitalization among 462 Spanish influenza survivors determined by the Cox proportional hazards model, Japan, 1919–1920*

Risk factor	Hazard ratio (95% CI)	p value
Diphasic fever	1.73 (1.33–2.25)	<0.001
≥6 d of high fever from admission	1.70 (1.29–2.25)	<0.001
≥26 breaths/min on maximum respiration rate	1.57 (1.19–2.06)	<0.001
Adventitious discontinuous lung sounds	1.56 (1.17–2.07)	<0.001

*Diphasic fever, a body temperature >38°C at a time after the initial fever had decreased to <37.5°C; high fever, body temperature >38°C.

## Conclusions

The length of hospitalization of patients with acute infectious diseases, including Spanish influenza, is associated with disease severity. Otherwise healthy soldiers who became patients during the second wave of Spanish influenza in Japan during 1919–1920 were severely affected. Adventitious discontinuous lung sounds, rapid respiration rate, and time-course of fever reflected disease severity during the pandemic. Patients who were severely affected had mainly fulminating fatal cases or experienced secondary bacterial pneumonia.

Severe disease associated with the recent pandemic caused by influenza A(H1N1)pdm09 virus can be attributed to viral pneumonia, superimposition of bacterial pneumonia, or other underlying conditions (*4*–*6*). Similar factors were likely responsible for severe disease associated with Spanish influenza, despite differences in the viruses themselves and available medical interventions. Therefore, viral pneumonia, superimposed bacterial pneumonia, and underlying conditions were likely associated with the length of hospitalization of our study subjects. Of the patients hospitalized for >21 days, 159 (93.5%) had severe respiratory symptoms, 123 (72.8%) experienced >6 days of continuous high fever, and most variables examined in this study were significantly higher in accordance with the longer days of the hospitalization group (Table 1). The cause of Spanish influenza was unknown at the time, but viral pneumonia may have developed in the early stages of infection among some patients (*7*–*9*). Viral pneumonia cannot be easily resolved without antiviral agents, as observed among patients with A(H1N1)pdm09 virus infection (*10*,*11*). Another potential explanation for an extended period of high fever is that time is required for eliminating the virus from the body (*5*).

Among the survivors, the respiration rate for patients hospitalized for >21 days was significantly higher than that of those who required shorter hospital stays (p<0.001) (Table 1). In addition, a high respiration rate was a risk factor for a lengthy hospital stay (Table 2). According to current medical practice, respiration rate is one of the consistent indicators for CURB-65 (a clinical prediction rule that has been validated for predicting mortality in community-acquired pneumonia by the British Thoracic Society [*12*]) and for severity on the pneumonia severity index of the American Thoracic Society/Infectious Disease Society of America (*13*).

Auscultation of the lungs remains the most useful examination technique for assessing airflow through the tracheal-bronchial tree. Patients with adventitious sounds experienced a longer hospital stay (Table 1). Adventitious discontinuous sounds were a significant risk factor for a lengthy hospitalization (Table 2), suggesting that pneumonia had developed in these patients. Additionally, bronchial sounds, rather than vesicular sounds audible at the chest wall, of the study patients suggest more severe pathophysiologic lung conditions such as severe pneumonia, pulmonary infarction, pulmonary massive bleeding, and diffuse alveolar damage, which was originally reported by Goodpasture (*7*,*8*). Bronchial sounds were more common among patients who died than among survivors (Table 1). Therefore, severe viral pneumonia may have developed in these patients, and they died shortly after disease onset.

Bacterial pneumonia has been previously reported as a common cause of death among Spanish influenza patients, especially during the second wave of illness (*8*,*14*). Samples from few patients in the study group underwent microbiologic examination. Thus, a diphasic fever might be explained by development of secondary bacterial pneumonia during hospitalization. Patients who experienced diphasic fever and a lengthy hospital stay were believed to have contracted a nosocomial infection (*8*,*9*,*14*).

Although patients with underlying diseases in the influenza pandemic of 2009 exhibited 1 of our identified 3 major prognostic indicators (*4*–*6*), we did not observe this pattern in the patients in our study, who were young soldiers and otherwise healthy and robust. At the time of the Spanish influenza pandemic, specific diagnostic methods and suitable techniques for evaluating disease severity were not available. Laboratory tests and specific treatment options were limited. The preserved medical charts emphasize the importance of providing information on physical assessments, such as body temperature, respiration rate, and lung sounds, to predict disease prognosis.

This study required review of charts handwritten ≈100 years ago, and some areas were unreadable. Because the patients were mainly young male soldiers, generalization of these results to the wider population in Japan who had Spanish influenza is limited. However, the historical documents containing this information are rare, and our results provide valuable information relevant for current and future clinical care of patients affected by pandemic influenza.

Patients experiencing influenza require rapid assessment of disease severity and prompt treatment. Our findings reveal conveniently assessed parameters to aid clinical decision-making, including triage, especially during the rampant stage of pandemic influenza, regardless of the medical resources available.
